# The Potential of mHealth as a Game Changer for the Management of Sickle Cell Disease in India

**DOI:** 10.2196/25496

**Published:** 2021-04-13

**Authors:** Ravindra Kumar, Aparup Das

**Affiliations:** 1 ICMR-National Institute of Research in Tribal Health Jabalpur India

**Keywords:** sickle cell disease, drug adherence, mHealth, India

## Abstract

Sickle cell disease (SCD) is a chronic genetic disease that requires lifelong therapy and monitoring. Low drug adherence and poor monitoring may lead to an increase in morbidities and low quality of life. In the era of digital technology, various mobile health (mHealth) apps are being tested for their potential in increasing drug adherence in patients with SCD. We herewith discuss the applicability and feasibility of these mHealth apps for the management of SCD in India.

## Background

In recent years, a revolution in information technologies has greatly influenced health care practices under the broad definition of digital health. Digital health practices are becoming highly adaptable in both developed and developing countries [1]. Moreover, with the increasing use of smartphones, smart watches, and artificial intelligence–based devices, mobile health (mHealth) is expected to define the standard of health care delivery across the globe. Specifically, mHealth apps can be used to increase disease awareness, increase drug adherence, provide cognitive behavioral therapies, and track health care delivery [2-5]. There are more than 325,000 mHealth apps available for Android and Apple smartphones [6]. Various mHealth apps have been clinically tested for their effect on compliance for many chronic diseases worldwide [7-11] and there are mounting indications that support the feasibility and applicability of mHealth interventions for better compliance in managing chronic diseases in pediatric patients as well. During the ongoing COVID-19 pandemic, mHealth has emerged as a silver bullet, not only for teleconsultations and telemonitoring of patients with chronic diseases, but also for increasing health care delivery in remote areas [12-14].

## Sickle Cell Disease: A Life-threatening and Highly Morbid Disorder

Sickle cell disease (SCD) is a genetic and chronic ailment, highly prevalent in Sub-Saharan Africa, the Middle East, the Mediterranean region, India, and parts of Central and South America. Globally, more than 300,000 children are born each year with SCD and three countries (Nigeria, India, and the Democratic Republic of Congo) bear about half of the global burden [15]. Patients with SCD often present with acute complications (eg, bone pain crisis, acute abdominal pain, acute chest syndrome, visceral sequestration crisis, aplastic crisis, acute anemia, cerebrocardiovascular complications, priapism). Chronic morbidities in SCD (eg, chronic pain syndromes, immunological and infectious complications, chronic lung disease, hepatobiliary complications, renal complications, leg ulcer, musculoskeletal complications, and psychosocial or psychiatric issues) are often encountered [16].

The under-five mortality of SCD varies significantly depending on the availability of health care facilities and infrastructure. For example, in low-income countries with poor access to health care services, mortality can reach up to 90% [15]. There is now growing evidence that continuous interventions through disease-modifying drugs such as hydroxyurea and prophylactic antibiotics can decrease morbidities and increase life expectancy, thereby leading to increased health-related quality of life and reduced health burden [17-22]. However, the sustainability of drug adherence is a major challenge in public health, as an average of about 50% of patients with chronic illness (including SCD) do not adhere to proper treatment in developed countries [23,24]. Furthermore, the prevalence of medication nonadherence is much higher in developing countries due to their relative scarcity and inequities of health care resources in comparison to developed nations [25-27]. Low drug adherence not only is associated with increased morbidity and poor health-related quality of life for patients, but also increases burden on the health economy and increases health care utilization in a country setting.

Various factors govern drug adherence, including delivery of health care services, economic situation, and cultural factors of patients [28]. Behavioral factors like forgetfulness, inappropriate time management, lack of awareness of disease, and fear of drugs pose additional barriers to drug adherence [29-31]. It has been observed that parents of patients with SCD with mild symptoms are less willing to accept the risk associated with taking hydroxyurea, particularly with regard to the long-term side effects, which include birth defects and cancer [29]. Therefore, patients with mild symptoms are more unwilling to take medications. In addition, patients with poor drug adherence can experience a mild-to-moderate medication response, causing frustration in both patients and their parents, which further leads to increased drug nonadherence.

Low drug adherence in SCD is also a major issue in India, where the majority of patients with SCD belong to scheduled communities (both the scheduled caste and scheduled tribes) [32,33], who face many barriers in accessing quality health care services. The scheduled caste and scheduled tribes are groups of people scheduled in the Constitution of India on the basis of economic, social, and educational disadvantage. Tribes are the most marginalized communities and mostly live in hard-to-reach remote hilly and forested areas. Many of the tribal communities are still dependent upon hunting and gathering and primitive agricultural practices. Poor accessibility, social disconnection, inconvenient timing, longer waiting times in government health care facilities, and poor economic conditions are some of the major roadblocks to accessing quality health care services in tribal areas in India [34]. Further, due to the different environments and terrains in which tribes live, inequalities in sociocultural behavior, and lack of participation, a universal design of health care services becomes inappropriate in tribal communities. Various nongovernmental organizations (NGOs)—such as SEARCH (in Gadchiroli, Maharashtra, India) [35], MAHAN (Melghat, Maharashtra) [36], Seva Rural (Jhagadia, Gujarat) [37], Jan Swasthya Sahyog (Ganiyari, Chhattisgarh) [38], and ASHWINI (Gudalur, Tamil Nadu) [39]—have developed different locally appropriate customized models for providing better health care services in hard-to-reach remote tribal areas.

With regard to SCD, various control programs have been in operation in India for an extended period of time. However, due to the lack of an organizational referral system and standard treatment guidelines for the management of SCD, most of these programs are limited to the screening of patients [40]. It has been observed that most patients seek treatment only when they have an acute sickle cell crisis. Moreover, the low accessibility and affordability of drugs is a major obstacle in the management of SCD in Indian tribes [41,42].

## The Role of mHealth in SCD Management: Indian Context

Disease interventions using mHealth have been tried for several diseases in tribal and rural India. For example, in a recent clinical trial, accredited social health activists (ASHAs) who used an mHealth app (ImTecho) as a job aid were able to provide better maternal and child health services in tribal areas in the state of Gujarat [43]. Similarly, presumptive tuberculosis referrals increased when rural health care providers used mHealth technology in tribal areas of Khuntu District of Jharkhand state [44]. Based on different models, a framework of digital health for increasing referrals, monitoring patients, and increasing the accessibility of malaria drugs in rural areas has been suggested [45]. A similar approach can be adopted for increasing drug availability and improving the referral system for SCD as well. Intervention measures (eg, sending reminders, allowing pain and symptom reporting, enabling self-management, and providing cognitive behavioral therapy) through mHealth apps for increasing drug adherence for SCD have been tested in several parts of the globe and have shown promising results in terms of disease outcome [46-48]. In addition, self-management practices in SCD have been found to be increased by the use of mHealth apps. However, no such intervention measures have previously been tried for patients with SCD in India. Considering SCD in India is mostly prevalent in scheduled communities living in rural and hard-to-reach areas, mHealth might be especially useful for SCD management. This is because access to health care services in hard-to-reach rural and forested areas remains meagre due to poor local transport systems. In these circumstances, mHealth apps can be used to improve the referral system in inaccessible areas by increasing knowledge among ASHAs, community health workers, and the traditional healers in the tribal communities.

**Figure 1:**
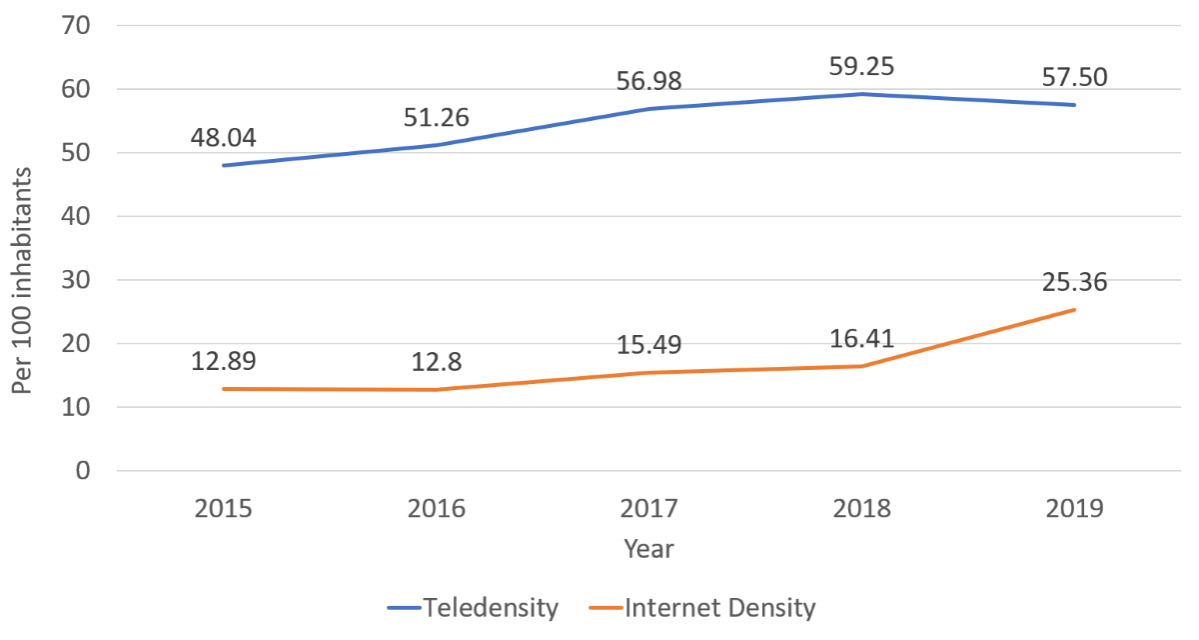
Telephone and internet density in rural India.

Is it practically feasible to use mHealth apps for patients with SCD in India? Several broad factors might limit the adoption of mHealth in patients with SCD in India (Textbox 1). One of the major roadblocks is poor internet availability in rural India. Although the number of mobile and internet users in rural areas is said to have increased substantially in recent years (Figure 1) [49], by the year 2018, just 14.9% of rural households had internet access [50]. Furthermore, mobile and internet connectivity are considered poor in hard-to-reach forested areas, which could hinder access to mHealth apps. In addition, the poor socioeconomic status of tribes further limits their ability to sustain the cost of smartphones and internet data plans for a longer duration. Moreover, a low level of digital literacy among rural and tribal people also impedes the usability of mHealth apps. Apart from this, people living in rural areas, especially tribes, have their own social beliefs and customs that are different from other populations in India, thereby limiting the use of such mHealth apps due to hesitation and stigma. In addition, privacy and cybersecurity issues related to the online use of any mobile app (including mHealth) may be a concern of people who are inadequately digitally literate and economically disadvantaged. Further, most mHealth apps require manual use; therefore, after a certain time period, patient engagement may decrease. Therefore, not only the feasibility, but also the long-term sustainability of mHealth apps in patients with SCD residing in rural and tribal areas is presently uncertain.

**Utility of and roadblocks to mobile health apps for the management of sickle cell disease in India.**

**Utility**

- Enhancement of referral system
- Strengthens public health delivery
- Augments drug adherence
- Creates disease awareness
- Improves self-management abilities during a primary sickle cell crisis

**Roadblocks**

- Low digital literacy
- Poor telecommunication connectivity in remote areas
- Cost of data plans for economically disadvantaged communities
- Digital privacy and data security

In spite of the above mentioned barriers to the use of mHealth apps in India, there is a light at the end of the tunnel. Based on the applicability of disease management and growing digitalization in India, measures should be taken to implement mHealth technology for SCD interventions with locally appropriate customized solutions. For example, mHealth apps with artificial intelligence that require little manual intervention may be helpful for patients with low digital literacy. In addition, for better management of SCD, user-specific customizable mHealth apps should be made, not only for increasing drug adherence but also for informing the patient about disease precipitating factors through daily activity monitoring and environmental conditions, thereby reducing acute crises. Such customization requires field-based and need-based approaches through direct inputs from patients as well as their caregivers in terms of expectations, needs, and experiences in combatting SCD to improve short-term engagement. Once such customized and user-friendly mHealth apps are in use by patients, regular feedback in terms of usage, technicality, and user-friendliness can be obtained from users at regular intervals for long-term sustainability. Furthermore, considering the poor economic conditions of many rural and tribal people, the provision of incentives (in term of free smartphones and internet data packs) may also serve as a boon for increased sustainability of mHealth apps for SCD intervention in India. In addition, encouragement through community engagement as part of a drive for increased use of mHealth apps could prove useful, as could the involvement of health ambassadors (eg, ASHAs, community health workers, community leaders, traditional healers, and/or adult SCD champions). These health ambassadors can be trained on the operation and use of mHealth apps, who in turn can train patients with SCD and their caregivers to use mHealth apps effectively. Moreover, socioeducational drives for increasing digital literacy among patients with SCD residing in rural and tribal areas can prove highly rewarding in terms of effective management. Furthermore, studies aiming to evaluate the cost effectiveness of mHealth interventions in improving health care delivery and drug adherence for patients with SCD living in remote areas are needed to assess the sustainability of such interventions.

## The Way Forward

In 2016, the Indian government aimed to increase the accessibility of hydroxyurea in district hospitals located in high-prevalence areas and issued guidelines for the prevention and control of hemoglobinopathies [51]. However, due to implementation gaps, the program could not be initiated in the majority of states, particularly in tribal areas [40]. Recently, a draft policy was prepared, which advocates for the improvement of SCD treatment centers in all districts and ensures a free supply of hydroxyurea and penicillin to low-income patients [52]. Furthermore, the recent introduction of the Ayushman Bharat scheme promulgating universal health coverage ensures free treatment of low-income patients admitted in hospitals [53]. For the successful implementation of these schemes, there is a pressing need for the development of an organizational referral system and a strong communication system for increasing awareness about diseases and the treatment and monitoring of patients. In this context, mHealth can appropriately establish itself as a game changer in the management of SCD in India. Therefore, the introduction of mHealth for SCD has huge potential in terms of enhancing health care delivery and management, and providing a better quality of life to patients globally, including in India. However, the feasibility, acceptability, and sustainability of such mHealth apps for the management of SCD in India is uncertain at present. For this, the keys to success are the following: a strong inclination from the government through health policy reform for socially and economically marginalized populations living in rural and hard-to-reach areas; a significant investment in infrastructure development for the strengthening of mobile and internet connectivity in these areas; and promulgation of digital literacy using intersectoral coordination and public-private partnerships in rural and tribal populations. The recent introduction of the Prime Minister’s Digital India Movement and the Prime Minister’s Rural Digital Literacy Movement—with a view to ensuring the availability of cost-effective high-speed internet to every citizen and empowering the rural population in the use of digital technologies, including the marginalized scheduled castes/tribes and differently abled persons—is one such welcome move in this direction. Furthermore, with advances in digital technologies, a reduction in the cost of internet data plans and a higher penetration of mobile and internet connectivity in rural and hard-to-reach areas are expected in the future. In light of the above developments, the future of mHealth in India seems bright, and this will lead to a better prognosis and improved health-related quality of life for patients with SCD.

## Abbreviations

ASHA: accredited social health activist
mHealth: mobile health
SCD: sickle cell disease
